# Supplementary material for: Aberrant phase separation and nucleolar dysfunction in rare genetic diseases
Source: Nature. 2023 Feb 8;614(7948):564–71. doi: 10.1038/s41586-022-05682-1 (PMC9931588; doi:10.1038/s41586-022-05682-1)
Supplement: Supplementary file 1 — This file contains Supplementary Note, Supplementary Figs. 1–6, Supplementary Table legends, Supplementary Video legends and supplementary references. [file 41586_2022_5682_MOESM1_ESM.pdf]

nature portfolio

https://doi.org/10.1038/s41586-022-05682-1

Supplementary information

Aberrant phase separation and nucleolar dysfunction in rare genetic diseases

In the format provided by the
authors and unedited

SUPPLEMENTARY INFORMATION

Aberrant phase separation and nucleolar dysfunction in rare genetic diseases

Martin A. Mensah[1,2,3,*], Henri Niskanen[4,*], Alexandre P. Magalhaes[4], Shaon Basu[4], Martin Kircher[5,6], Henrike L. Sczakiel[1,2,3], Alisa M. V. Reiter[1], Jonas Elsner[1], Peter Meinecke[7], Saskia Biskup[8], Brian H. Y. Chung[9], Gregor Dombrowsky[10,11], Christel Eckmann-Scholz[12], Marc Phillip Hitz[10,11], Alexander Hoischen[13,14], Paul-Martin Holterhus[15], Wiebke Hülsemann[16], Kimia Kahrizi[17], Vera M. Kalscheuer[3], Anita Kan[18], Mandy Krumbiegel[19], Ingo Kurth[20], Jonas Leubner[21], Ann Carolin Longardt[22], Jörg D. Moritz[23], Hossein Najmabadi[17], Karolina Skipalova[1], Lot Snijders Blok[14], Andreas Tzschach[24], Eberhard Wiedersberg[25], Martin Zenker[26], Carla Garcia-Cabau[27], René Buschow[28], Xavier Salvatella[27,29], Matthew L. Kraushar[4], Stefan Mundlos[1,3,30], Almuth Caliebe[6], Malte Spielmann[3,6,31,32,$], Denise Horn[1,32,$], Denes Hnisz[4,32,$]

[$] Correspondence:

hnisz@molgen.mpg.de

Denise.Horn@charite.de

Malte.Spielmann@uksh.de

CONTENTS

- Supplementary Notes
- Supplementary Figures 1-6
- Supplementary Table Legends
- Supplementary Video Legends
- Supplementary References

Supplementary Tables 1-6, and Supplementary Videos 1-2 are uploaded separately.

Supplementary Notes

Phenotypic and clinical findings of BPTAS individuals (I1-I5), and I6

Individual 1

Individual 1 (I1) affected with BPTAS is a 9 months old boy (**Fig. 1a-b, Extended Data Fig. 1a-c, Supplementary Table 1**) born to healthy non-consanguineous parents (maternal age 21 years, paternal age 36 years) of German descent, after 38+5-weeks of gestation with a weight of 1825 g (-3.4 SD), a length of 33 cm (- 7.8 SD), and an occipitofrontal head circumference (OFC) of 28 cm (- 4.9 SD). The family history was unremarkable. At the 21st week of pregnancy, prenatal ultrasound examination revealed microcephaly, short limbs with missing tibiae, dysplastic hip bones, brachydactyly of the hands, and polysyndactyly of the feet. Postnatally, a cleft in the midline of the upper jaw, webbed elbow joints with a pit on the left side, ambiguous genitalia including a micropenis and cryptorchidism as well as dysplastic nails of the feet were found. Facial dysmorphism included hypertelorism, narrow palpebral fissures, a short nose with a flat nasal bridge, microtic low-set ears, a pre-auricular skin tag on the right, and a small chin. At newborn age radiograms of upper and lower limbs showed disproportionate shortening of the radius and ulna, dislocation of the radial head, webbed elbows, brachydactyly/brachyphalangy, tibial aplasia, hypoplasia of fibulae, club feet, and preaxial polysyndactyly. Examination at the age of 9 months revealed microcephaly with an OFC of 38.5 cm (-5.3 SD), and a length of 44 cm (-10 SD). At this age, he presented with global developmental delay and hearing impairment.

Individual 2

Individual 2 (I2) affected with BPTAS is a 29-year-old female with healthy non-consanguineous parents of German and Kirgisian descent (**Fig. 1a-b, Extended Data Fig. 1d-i, Supplementary Table 1**). After birth, a babygram showed tibial aplasia, hypoplastic/absent fibulae, hypoplastic pelvic bones, contractures of joints, and hypoplasia of the right femur. Examination revealed brachydactyly of the hands with dysplastic nails and polysyndactyly of the feet. Furthermore, she showed microcephaly, microtia with dysplastic pinnae, blepharophimosis, high palate, and a short neck. In addition, she had moderate intellectual disability and conductive hearing impairment. Gastro-esophageal reflux was treated with a fundoplication at the age of 11 years. The parents of I2 were not available for genetic testing.

Individual 3

Individual 3 (I3) is an Iranian girl affected with BPTAS originally described in a previous study [1] (**Supplementary Table 1**).

Individual 4

Individual 4 (I4) affected with BPTAS is a 11-month-old boy born to healthy non-consanguineous (maternal and paternal age at birth 27 years) parents of German descent, after 39+1 weeks of gestation with a weight of 2320 g (-2.73 SD), a length of 46 cm (-2.6 SD), and an OFC of 29 cm (-4.88 SD) (**Fig. 1b**, **Extended Data Fig. 1j-l, Supplementary Table 1)**. The family history was unremarkable. Prenatally, an intrauterine growth retardation was noted. Prenatal ultrasound also suggested club feet which prompted an amniocentesis with chromosomal analysis which showed a normal male karyotype. Postnatal audiometry was unremarkable. Examination at the age of 12 weeks showed microcephaly with an OFC of 38 cm (-2.12 SD), a weight of 5230 g (-0.63 SD), and a length of 56 cm (-1.43 SD). Craniofacial dysmorphism included hypertelorism with blepharophimosis, a thin upper lip vermillion, deeply set, microtic ears, a nuchal nevus flammeus, and a short neck. Both upper and lower limbs were shortened with unusually patterned palmar and dorsal creases of the hands and preaxial polysyndactyly, cutaneous syndactyly and abduction contractures of the feet. At age of 6 months, radiograms of upper and lower extremities showed brachydactyly/brachyphalangy, slightly asymmetric shortness of tibiae and fibulae as well as delayed epiphyseal maturation. A deep and broad sacral dimple and hypospadias were noted. Intermittently dropping blood oxygen levels required pulse oximetry monitoring during the first 10 weeks of life.

Individual 5

Individual 5 (I5) is a female fetus (**Fig. 1a-b, Extended Data Fig. 1m-r, Supplementary Table 1**) briefly reported as having a complex limb phenotype in a cohort of individuals with various limb malformations [2]. We evaluated this phenotype as BPTAS. The family history was unremarkable. The fetus was conceived by a healthy non-consanguineous Chinese couple (maternal age 32, paternal age 36). Ultrasound examination revealed bilateral absence of the tibia, head circumference at the 3rd percentile, and ambiguous external genitalia. The pregnancy was terminated after 21 weeks of gestation. Examination of the fetal face showed a flat nasal bridge, hypertelorism, small, low-set, dysplastic ears and a small mouth. There were multiple pterygia of the neck, of both shoulders and of the elbows. Examination of the hands

revealed short fingers and thumbs with bulbous tips. There was a bony sacro-coccygeal prominence with a patent anus, and ambiguous external genitalia. A fetogram revealed brachydactyly/brachyphalangy, abduction contractures of the hips, hypoplastic pelvic bones, absence of the tibia, hypoplastic and curved fibulae, and mirror-image polysyndactyly. In addition, horseshoe kidneys were found.

Individual 6

Individual 6 (I6) is the first child of healthy non-consanguineous parents of Venezuelan descent (**Supplementary Table 2**). He was delivered after 40 weeks of gestation with a birth weight of 2770 g (-1.22 SD), and a birth length of 49 cm (-1.06 SD). His OFC at day 14 was 35 cm (mean). He was able to sit without support at the age of 6 months and to walk at 18 months. At the age of 5 years and 7 months, his height was 102 cm (-2.79 SD), his weight 17 kg (-1.5 SD), and his OFC 48 cm (-3.08 SD). He displayed a delay in speech development and was using short sentences but with grammatical errors. He presented with unilateral ptosis, a short neck, clinodactyly of the fifth fingers, and syndactyly of the second and third toes. The Snijders-Oomen non-verbal intelligence test resulted in an IQ score of 67. Ophthalmological examination showed hyperopia and astigmatism. Chromosome analysis showed a normal male karyotype. Chromosomal microarray analysis (CMA), however, identified the heterozygous microdeletion arr[hg19]13q12.3(31,035,934_31,117,112)x1 affecting at least exons 1 to 4 of HMGB1 (**Extended Data Fig. 2h-j**). qPCR revealed a deletion of the entire gene and showed a de novo occurrence (**Extended Data Fig. 2j**).

Clinical comparison of individuals affected with BPTAS

The phenotype of the five affected individuals analyzed here (I1-5) and the eight others described in the literature is highly specific and comprises a distinct and uniform pattern of skeletal anomalies including tibia aplasia/hypoplasia, preaxial polysyndactyly of the feet, and brachyphalangy of the fingers as well as consistent craniofacial features comprising malformed ears/microtia as well as hypertelorism/telecanthus and short palpebral fissures which was confirmed by computer-aided facial analysis (**Fig. 1a-b, Supplementary Table 1, Extended Data Fig. 1s**) [1,3-8]. Hand radiograms in I1-I3, I5 and those reported in the literature display a uniform pattern of brachyphalangy including short middle phalanges and short proximal phalanges of the thumb. Hand radiogram of I4 is milder affected and showed short tubular bones with mildly more shortness of the middle phalanges. (**Fig. 1b, Supplementary Table 1**). Hypoplastic/absent nails combined with lateral dislocation of some rudimentary nails and

enlargement of distal phalanges in the dorso-ventral axis documented in almost all affected individuals were interpreted as dorso-ventral patterning defects (**Fig. 1a, Extended Data Fig. 1b, Supplementary Table 1**). In addition to the main skeletal anomalies, contractures/pterygia are present in the majority of affected individuals. The further phenotypic spectrum of BPTAS is variable: hypoplastic pelvic bones were documented in almost all affected individuals with exception of I4 reported here. The tibial involvement varies from absence to shortness. Microcephaly and genitourinary anomalies such as hypoplasia of the scrotum and labia belong to the frequent features (**Supplementary Table 1**). The majority of affected individuals were found to have hearing impairment (**Supplementary Table 1**). A motor or psychomotor delay was noted in some of them (**Supplementary Table 1**).

Genotype-phenotype correlation of *HMGB1* variants

Microdeletions of chromosome 13q12.3 - which involves *HMGB1* - cause a syndrome characterized by developmental delay and microcephaly (**Supplementary Table 2**) [9-13]. A recent study revealed that the critical region of this microdeletion syndrome covers only one protein-coding gene: *HMGB1*, and that loss of function variants of the first four exons of *HMGB1* may cause the core phenotype of 13q12.3 deletion syndrome [9]. The de novo deletion of *HMGB1* in I6 in our study supports these previous findings that loss of *HMGB1* function leads to a neurodevelopmental disorder [9].

Regarding the skeletal involvement there are distinct clinical spectra of BPTAS and of individuals with 13q12.3 microdeletions or loss of function *HMGB1* variants. However, microcephaly is present in both entities and the majority of BPTAS individuals with available corresponding data was documented as having a developmental delay. We suggest that the different clinical manifestations of the two syndromes are likely caused by the different types of *HMGB1* variants found. Loss of function variants reported to be pathogenic are found in *HMGB1*'s first four exons and are thus likely to result in nonsense-mediated decay (NMD), i.e. they are amorphic [9]. The same mechanism is suggested for the deletions described in 13q12.3 deletion syndrome. The only missense variant classified as pathogenic c.329G>A;p.(Arg110His) as well as the only frameshift variant that might escape NMD c.437dupA;p. Lys147Glufs*10) reported in individuals with neurodevelopmental anomalies affect the protein's HMG-box-2 domain [9]. In contrast, the frameshift variants identified in individuals with BPTAS affect HMGB1's acidic tail encoded by the gene's final exon (**Fig. 1c**). Both wild type and mutant RNA transcripts were detectable in LCL cells of I3, suggesting the BPTAS-causing frameshift variants

likely do not lead to NMD (**Extended Data Fig. 2d**). Neurodevelopmental delay and microcephaly shared by both HMGB1-associated phenotypes are unspecific signs seen in a variety of genetic syndromes. A common molecular etiology of these symptoms in the two phenotypes is thus neither confirmed nor precluded. Nevertheless, our data suggest that the skeletal aspects of BPTAS are specifically result from frameshifts altering HMGB1's acidic tail.

While our data suggest altered nucleolar and nuclear properties of HMGB1 mutant variants featuring a replaced tail domain, no functional data on *HMGB1* mutant variants associated with the neurodevelopmental phenotype have been reported. Nevertheless, as the neurodevelopmental phenotype is apparently caused by *HMGB1* loss of function variants, these variants - in contrast to the C-terminal frameshifts causing BPTAS - do not alter HMGB1 function but are likely causing pathogenic dosage effects.

Nucleolar effects of the frameshift HMGB1 variants described here suggest that BPTAS could be classified as a ribosomopathy. As BPTAS and other ribosomopathies directly affect cell growth and proliferation, they are all associated with a broad spectrum of malformations and craniofacial anomalies. However, these phenotypic features are unspecific, seen in a variety of inheritable malformation syndromes. The characteristic combination of skeletal findings of BPTAS (e.g. the missing/hypoplastic tibiae, preaxial foot polydactyly, brachyphalangy/brachydactyly of hands, unique facial gestalt) clearly distinguishes BPTAS from other ribosomopathies such as Diamond-Blackfan anemia or Treacher-Collins syndrome. Interestingly, BPTAS and Treacher-Collins syndrome share microtia. No hematological or malignant findings are described in BPTAS compared to Diamond-Blackfan anemia and other ribosomopathies.

Clinical review of genes in the mutation catalog

101 pathogenic frameshift variants that create an arginine-rich tail in 66 genes were identified in the mutation catalog (**Fig. 4a, Supplementary Table 5**). 13 of these genes were associated with an autosomal dominant disease, although the affected gene's pLI was lower than 0.05 suggesting a possible gain of function effect of the associated variants. 10 genes were associated with two or more clinically distinct phenotypes (*POLE, POMGNT1, PRX, SQSTM1, SYNE1, NKX2-1, PDZD7, TP53, FOXC1, PTCH1*). Among these were *FOXC1* which is associated with anterior segment dysgenesis and CAKUT, *SQSTM1* associated with IBMPFD4 and childhood-onset neurodegeneration, *PTCH1* associated with both Gorlin-Goltz syndrome

and holoprosencephaly 7 [14] , and *TP53*. *TP53* is especially interesting as loss of function variants cause the tumor predisposition Li-Fraumeni syndrome while (recurrent) de novo frameshifts altering the proteins C-terminal tail cause bone marrow failure syndrome 5 characterized by severe growth retardation, testicular atrophy, delayed bone age, neurodevelopmental delay and red cell aplasia [15,16].

Of the 103 pathogenic frameshift variants (57 genes) in the catalog that add a hydrophobic patch to the mutated protein 38 variants affect one of 24 genes with a pLI lower than 0.05. 7 of these genes are associated with an autosomal dominant phenotype (*AIRE, ASXL1, ELN, FOXL2, ITGA2B, TBC1D24, MYRF*). Among these were *ASXL1* associated with Bohring-Opitz-syndrome, *FOXL2* associated with *BPES* (discussed below) and *ELN*. Notably, loss of function variants of *ELN* including deletions of the entire gene are associated with supravalvar aortic stenosis, while specific C-terminal variants including the frameshift NM_000501.4(*ELN*):c.2058del;p.(Gly688fs) result in cutis laxa [17,18]. 12 genes (including *ELN*) featuring frameshifts in ClinVar that add hydrophobic patches to the C-terminus of the mutated protein are associated with at least two clinically distinct phenotypes on OMIM. Notably, C-Terminal frameshifts of *MYRF* including NM_001127392.3(MYRF):c.3361del;p.(Arg1121fs) cause autosomal dominant nanophthalmos while various mutations of other parts of the gene result in cardiac-urogenital syndrome or mild encephalopathy with reversible myelin vacuolization [19,20].

Genotype-phenotype correlations of selected variants' genes

HMGB3

An inherited frameshift in the final exon of *HMGB3* has been linked to syndromic microphthalmia type 13 [21]. HMGB3 shows strong sequence similarity with HMGB1 and intriguingly the causative variant in HMGB3 results in the replacement of the HMGB3 protein's acidic tail as seen in our study of frameshifts changing HMGB1's acidic tail. No other variant in *HMGB3* has yet been linked to a disease. The syndrome is potentially rare as only the specific replacement of HMGB3's acidic tail causes microphthalmia, just as specific frameshift of HMGB1's acidic tail but no other variants in HMGB1 cause BPTAS.

MYOD1

Biallelic pathogenic germline variants of MYOD1 are associated with only one inheritable

disease, a syndromic form of myopathy [22].

RAX

Biallelic germline mutations of *RAX* are associated with isolated microphthalmia type 3 [23]. No other syndrome is linked to *RAX*.

FOXC1

Monoallelic germline mutations of *FOXC1* cause anterior segment dysgenesis of the eye or congenital anomalies of the kidney and urinary tract (CAKUT). Truncating variants and those affecting the ordered FH domain of FOXC1 result in eye anomalies while CAKUT appears to be caused by non-truncating variants outside of the FH domain (i.e. in the intrinsically disordered part of the protein) [24].

FOXF1

Pathogenic, heterozygous germline variants of *FOXF1* are so far associated with only one disorder: alveolar capillary dysplasia with misalignment of pulmonary veins (ACD/MPV) [25].

RUNX1

Germline monoallelic pathogenic variants of RUNX1 result in an inheritable platelet disorder with a predisposition to myeloproliferative diseases, somatic mutations have also been described in hematopoietic diseases [26].

SQSTM1

Heterozygous pathogenic germline variants in *SQSTM1* result in a multisystemic phenotype of variable expressivity affecting the nervous system, muscles and bones. Original assumptions that symptoms of different organ systems represented separate disease entities caused by the same gene were challenged, when identical variants were found in individuals diagnosed with different entities. The autosomal-dominant SQSTM1-related disorder is probably best described as inclusion body myopathy with early-onset Paget disease with or without frontotemporal dementia type 4 (IBMPFD4; also denoted as multisystem proteinopathy type 4, MSP4) [27,28]. Biallelic pathogenic variants of *SQSTM1* are associated with a childhood-onset neurodegeneration [29,30].

FOXL2

Three phenotypes are associated with monoallelic, pathogenic germline variants of *FOXL2*: isolated premature ovarian failure (iPOF) and the two types of blepharophimosis-ptosis-epicanthus inversus syndrome (BPES), type 1 with ovarian failure, type 2 without ovarian failure [31]. It has been suggested that truncations downstream of the protein's polyalanine stretch result in ovarian failure (iPOF or BPES1), while those upstream cause BPES2. Due to intrafamilial phenotypic variability a clear phenotype-genotype correlation could not yet be confirmed [32,33].

PHOX2B

Heterozygous pathogenic germline variants of *PHOX2B* result in congenital central hypoventilation syndrome (CCHS), with or without Hirschsprung's disease and neural crest tumors. Most cases are caused by expansions of the protein's polyalanine repeat stretch, severity of the disorder appears to be roughly positively correlated with the length of the expansion. Disease severity ranges from sporadic neuroblastoma to severe CCHS [34].

CALR

Pathogenic germline variants of *CALR* have not yet been described, somatic mutations were described in disorders of the hematopoietic system (myelofibrosis, thrombocythemia) [35].

SOX2

Heterozygous pathogenic germline mutations of *SOX2* result in a disorder characterized by micro- or anophthalmia, brain malformations and hypogonadotropic hypogonadism. The expressivity is variable even within families. The vast majority of causative variants are loss-of-function variants and no clear genotype-phenotype correlation is apparent [36].

MEN1

Pathogenic heterozygous germline variants in MEN1 have only been associated with one disorder autosomal-dominant multiple endocrine neoplasia type 1. Somatic mutations have been described in sporadic tumors [37].

DVL1

Heterozygous variants of *DVL1* result in autosomal dominant Robinow syndrome type 2 [38,39]. The disorder is characterized by a combination of mesomelic limb shortening with brachydactyly, broad toes and thumbs and hypoplastic distal phalanges, genital hypoplasia, a characteristic facial gestalt comprising macrocephaly, frontal bossing midface hypoplasia,

hypertelorism and dental anomalies, and increased bone density. Notably, only specific C-terminal heterozygous frameshift variants escaping NMD and altering the disordered tail of *DVL1* are pathogenic (**Supplementary Figure 5**). The minimal affected region of the C-terminus consists of >15% arginines, contains a nucleolar localization signal and a hydrophobic patch (**Extended Data Fig. 8**) Other variants including LoF variants (pLI = 0) exist but appear not to be linked to a disease.

Supplementary Figure 1

Uncropped gel images from Extended Data Figure 4b

Before SEC

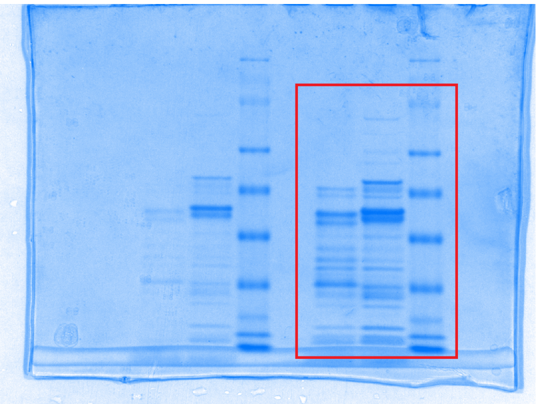

Mutant WT

After SEC

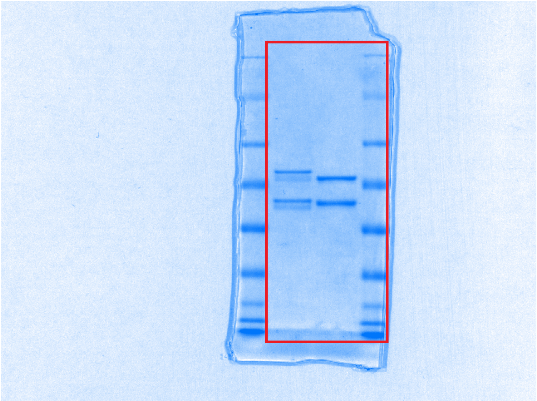

WT Mutant

Uncropped gel images from Extended Data Figure 4c

EGFP Antibody

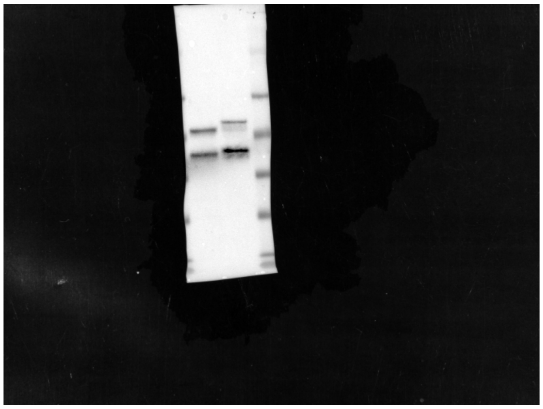

Mutant WT

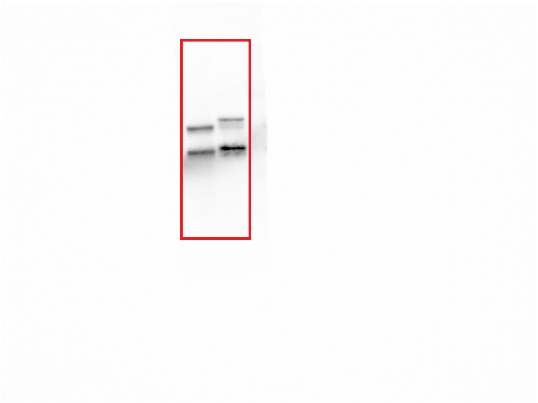

Mutant WT

HMGB1 Antibody

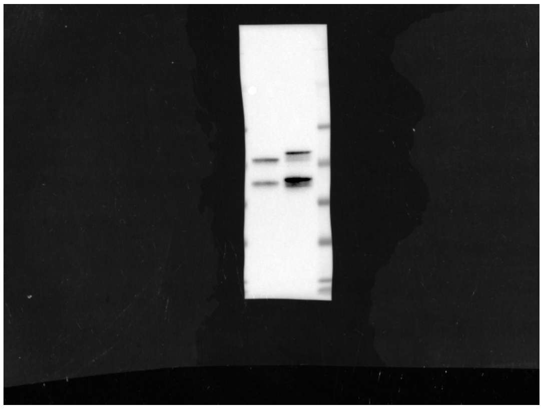

Mutant WT

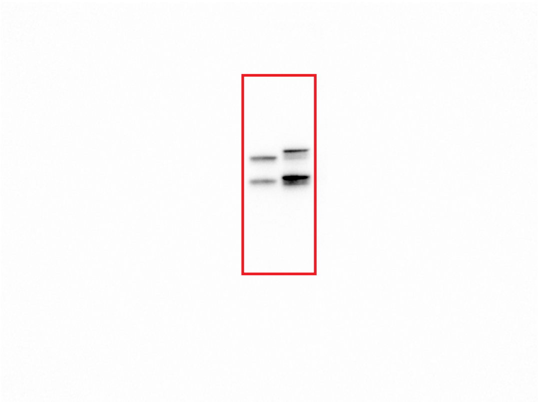

Mutant WT

Supplementary Figure 1. Source Data - uncropped gels and blots

Uncropped gel images and blot images of the data displayed in **Extended Data Fig. 4b-c.**

Supplementary Figure 2

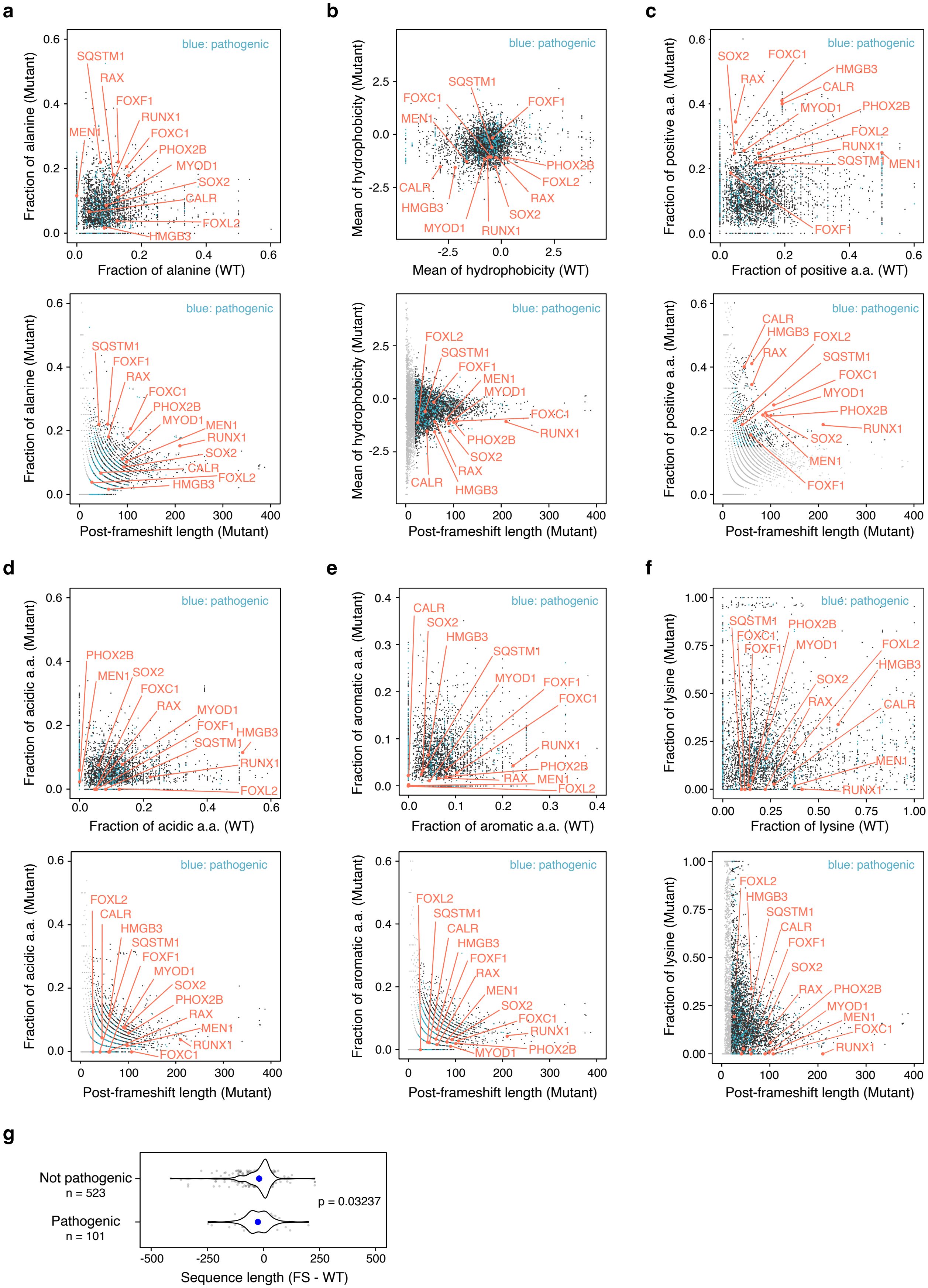

Supplementary Figure 2. Additional sequence features of sequences created by frameshift variants in C-terminal IDRs.

The figure panel (**a-f**) consist of a top and bottom subpanel. The top subpanels include the quantification of the fraction of indicated amino acids, or parameter in the mutant sequences created by frameshift variants on the y-axis, plotted versus in the wild type sequence replaced by the frameshift on the x-axis. Pathogenic variants are highlighted in blue. In the bottom subpanels, the fraction of indicated amino acids, or parameter in the mutant sequences created by frameshift variants is plotted against the length of the sequence created by the frameshift variant. Pathogenic variants are highlighted in blue.
(**a**) Alanines
(**b**) Mean hydrophobicity
(**c**) Positively charged amino acids
(**d**) Acidic amino acids
(**e**) Aromatic amino acids
(**f**) Lysines
(**g**) Comparison of the length differential of the arginine rich (≥20 amino acids, ≥15% arginine) pathogenic and non-pathogenic frameshifts. P-value is a Wilcoxon test.

Supplemetary Figure 3

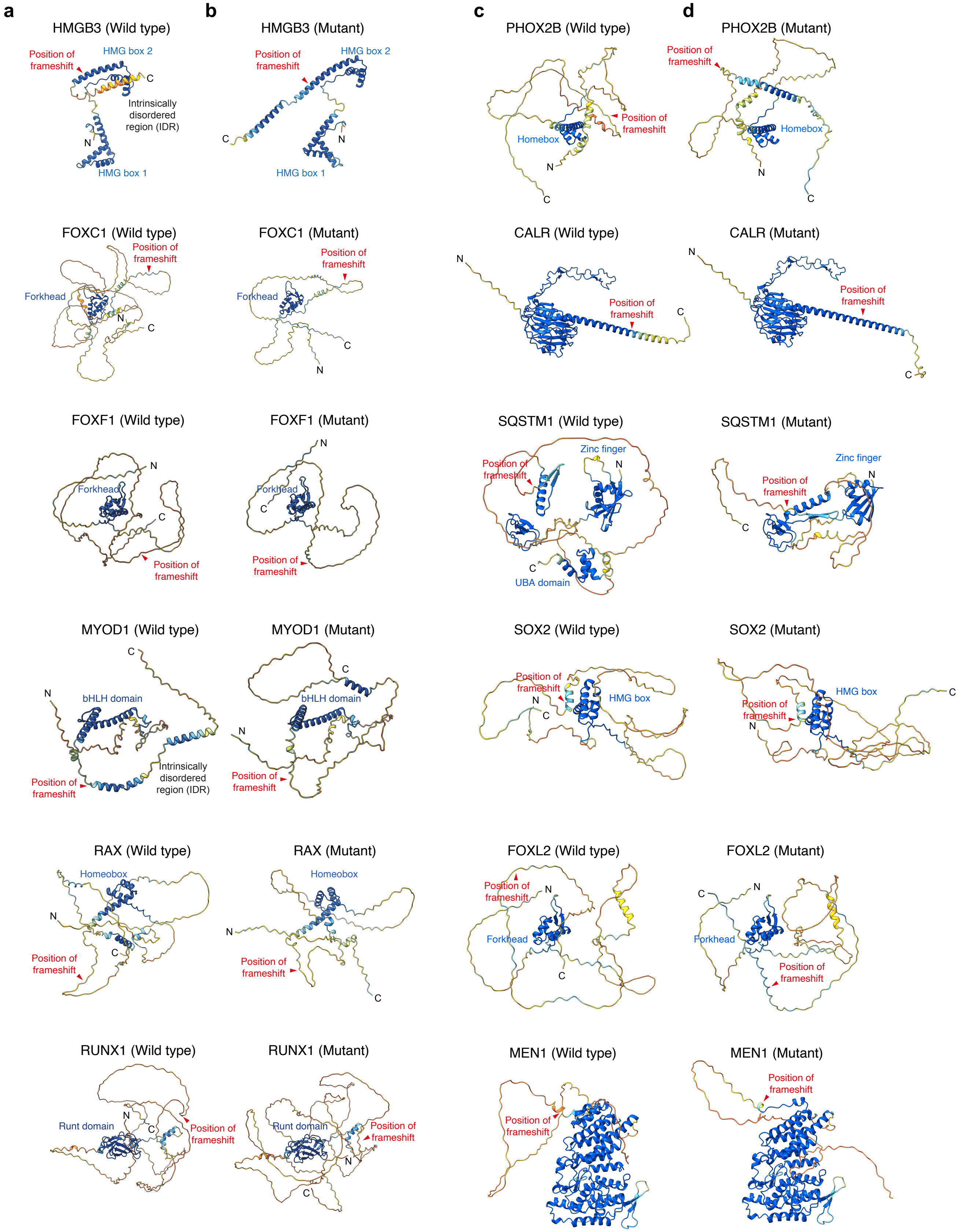

Supplementary Figure 3. AlphaFold2 models of wild type mutant proteins containing disease-associated frameshifts.

(**a**) Predicted structures of wild type HMGB3, FOXC1, FOXF1, MYOD1, RAX and RUNX1 proteins. Colors ranging from blue to orange depict the per-residue measure of local confidence for the model.
(**b**) Predicted structures of frameshift mutant HMGB3, FOXC1, FOXF1, MYOD1, RAX and RUNX1 proteins. Colors ranging from blue to orange depict the per-residue measure of local confidence for the model.
(**c**) Predicted structures of wild type PHOX2B, CALR, SQSTM1, SOX2, FOXL2 and MEN1 proteins. Colors ranging from blue to orange depict the per-residue measure of local confidence for the model.
(**b**) Predicted structures of frameshift mutant PHOX2B, CALR, SQSTM1, SOX2, FOXL2 and MEN1 proteins. Colors ranging from blue to orange depict the per-residue measure of local confidence for the model.

Supplementary Figure 4

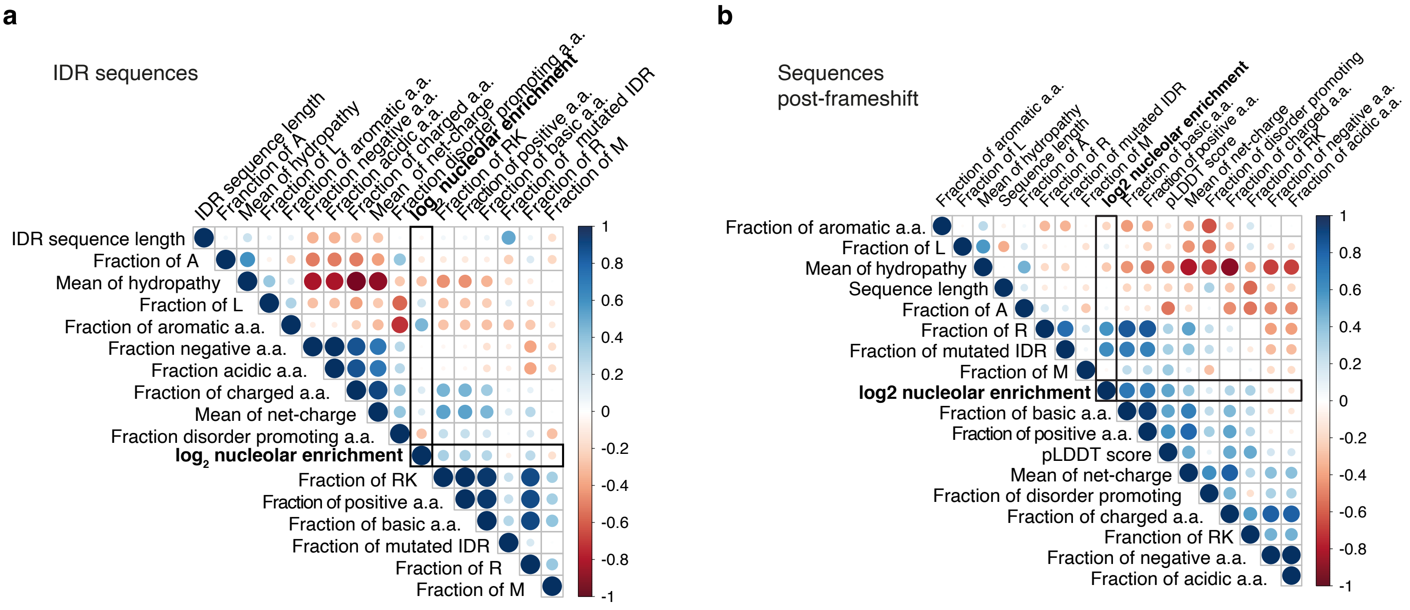

Supplementary Figure 4. Determinants of nucleolar enrichment of mutant proteins.

(**a**) Correlation analysis of the extent of nucleolar mispartitioning of mutant proteins (Fig. 4i) with various protein features of their IDRs. The color of the circles corresponds to the value of Pearson's correlation coefficients, and the size of the circles is proportional to the p-value of the Pearson's *r*. The features of the entire IDR are quantified for the correlation analysis. Note that the strongest correlation occurs between the extent of nucleolar mispartitioning and the fraction of wild type IDR removed by the frameshifts. A.a.: amino acid.
(**b**) Same as (a), except the quantified features of only the sequences created by the frameshifts were used for the correlation analysis. Note that the strongest correlation occurs between the extent of nucleolar mispartitioning and the fraction basic residues and the fraction of arginines in the sequences created by the frameshifts. A.a.: amino acid.

Supplementary Figure 5

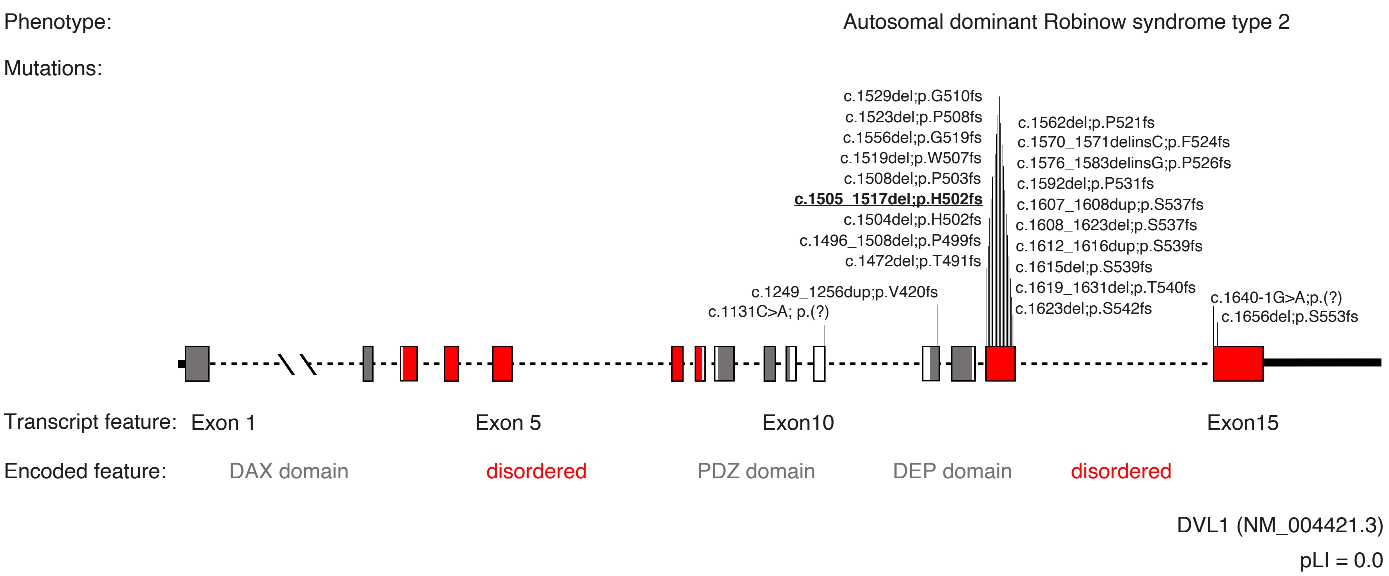

Supplementary Figure 5. DVL1 variants associated with Robinow syndrome

Position of frameshift variants in the disordered tail of DVL1, associated with autosomal dominant Robinow syndrome type 2. Structured domains are shown in gray and disordered regions predicted by PONDR VSL2 algorithm are shown in red.

Supplementary Figure 6

a

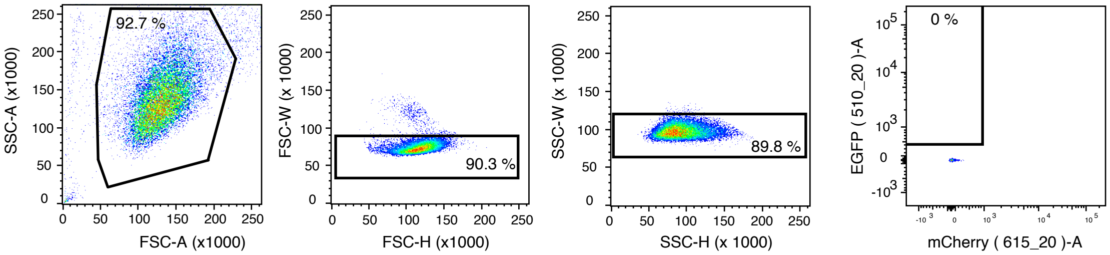

b

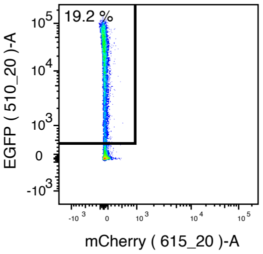

Supplementary Figure 6. FACS plots

Gating strategy to select for EGFP+ cells. Percentage of cells from total population selected by gating is shown on top of the figures. This gating strategy was used for collecting cells for Figure 3j, Extended Data Figure 6a, 6d, 6e, 6i, 6j, Extended Data Figure 10c, 10d.

(**a**) Example sort from untransfected (EGFP negative) cells.

(**b**) Selection of EGFP+ cells from mEGFP-HMGB1-WT transfected cells.

Supplementary Table Legends

Supplementary Table 1. Clinical characteristics of affected individuals with BPTAS
(Uploaded as separate word document.)

Supplementary Table 2. Clinical features of present and previously published individuals with 13q12.3 deletion syndrome
(Uploaded as separate excel sheet.)

Supplementary Table 3. Genes and isoforms whose protein products contain a C-terminal intrinsically disordered region (IDR)
(Uploaded as separate excel sheet.)

Supplementary Table 4. List of variants in C-terminal IDRs
(Uploaded as separate excel sheet.)

Supplementary Table 5. Features of proteins affected by frameshift variants, including sequence, length, pathogenicity, disease association
(Uploaded as separate excel sheet.)

Supplementary Table 6. Primer sequences
(Uploaded as separate excel sheet.)

Supplementary Video Legends

Supplementary Video 1. Fusion event between EGFP-HMG1 protein droplets *in vitro*. Time (mm:ss) is displayed on the bottom left. Scale bar = 1 µm.

Supplementary Video 2. Live U2OS cells expressing mutant EGFP-HMGB1 protein undergoing mitosis. Time after Doxycycline induction (hh:mm) is shown on bottom left of the image. Scalebar = 10 µm.

Supplementary References

1 Shafeghati, Y. *et al.* Brachyphalangy, polydactyly and tibial aplasia/hypoplasia syndrome (OMIM 609945): case report and review of the literature. *Eur J Pediatr* **169**, 1535-1539, doi:10.1007/s00431-010-1267-7 (2010).

2 Elsner, J. *et al.* Genome sequencing in families with congenital limb malformations. *Hum Genet* **140**, 1229-1239, doi:10.1007/s00439-021-02295-y (2021).

3 Baraitser, M. *et al.* A syndrome of brachyphalangy, polydactyly and absent tibiae. *Clin Dysmorphol* **6**, 111-121 (1997).

4 Faravelli, F., Di Rocco, M., Stella, G., Selicorni, A. & Camera, G. Brachyphalangy, feet polydactyly, absent/hypoplastic tibiae: a further case and review of main diagnostic findings. *Clin Dysmorphol* **10**, 101-103, doi:10.1097/00019605-200104000-00005 (2001).

5 Pierson, D. M. *et al.* Total anomalous pulmonary venous connection and a constellation of craniofacial, skeletal, and urogenital anomalies in a newborn and similar features in his 36-year-old father. *Clin Dysmorphol* **10**, 95-99, doi:10.1097/00019605-200104000-00004 (2001).

6 Olney, R. S. *et al.* Limb/pelvis hypoplasia/aplasia with skull defect (Schinzel phocomelia): distinctive features and prenatal detection. *Am J Med Genet* **103**, 295-301 (2001).

7 Wechsler, S. B., Lehoczky, J. A., Hall, J. G. & Innis, J. W. Tibial aplasia, lower extremity mirror image polydactyly, brachyphalangy, craniofacial dysmorphism and genital hypoplasia: further delineation and mutational analysis. *Clin Dysmorphol* **13**, 63-69, doi:10.1097/00019605-200404000-00002 (2004).

8 Bernardi, P. *et al.* Additional features in a new case of a girl presenting brachyphalangy, polydactyly and tibial aplasia/hypoplasia. *Am J Med Genet A* **149A**, 1532-1538, doi:10.1002/ajmg.a.32943 (2009).

9 Uguen, K. *et al.* Heterozygous HMGB1 loss-of-function variants are associated with developmental delay and microcephaly. *Clin Genet* **100**, 386-395, doi:10.1111/cge.14015 (2021).

10 Bartholdi, D. *et al.* A newly recognized 13q12.3 microdeletion syndrome characterized by intellectual disability, microcephaly, and eczema/atopic dermatitis encompassing the HMGB1 and KATNAL1 genes. *Am J Med Genet A* **164A**, 1277-1283, doi:10.1002/ajmg.a.36439 (2014).

11 Firth, H. V. *et al.* DECIPHER: Database of Chromosomal Imbalance and Phenotype in Humans Using Ensembl Resources. *Am J Hum Genet* **84**, 524-533, doi:10.1016/j.ajhg.2009.03.010 (2009).

12 Mandrile, G. *et al.* A new case of 13q12.2q13.1 microdeletion syndrome contributes to phenotype delineation. *Case Rep Genet* **2014**, 470830, doi:10.1155/2014/470830 (2014).

13 Wang, M., Li, B., Liao, Z., Jia, Y. & Fu, Y. A novel phenotype of 13q12.3 microdeletion characterized by epilepsy in an Asian child: a case report. *BMC Med Genomics* **13**, 144, doi:10.1186/s12920-020-00801-1 (2020).

14 Reinders, M. G. *et al.* New mutations and an updated database for the patched-1 (PTCH1) gene. *Mol Genet Genomic Med* **6**, 409-415, doi:10.1002/mgg3.380 (2018).

15 Toki, T. *et al.* De Novo Mutations Activating Germline TP53 in an Inherited Bone-Marrow-Failure Syndrome. *Am J Hum Genet* **103**, 440-447, doi:10.1016/j.ajhg.2018.07.020 (2018).

16 Fedorova, D. *et al.* De novo TP53 germline activating mutations in two patients with the phenotype mimicking Diamond-Blackfan anemia. *Pediatr Blood Cancer* **69**, e29558, doi:10.1002/pbc.29558 (2022).

17 Tassabehji, M. *et al.* An elastin gene mutation producing abnormal tropoelastin and abnormal elastic fibres in a patient with autosomal dominant cutis laxa. *Hum Mol Genet* **7**, 1021-1028, doi:10.1093/hmg/7.6.1021 (1998).

18 Hadj-Rabia, S. *et al.* Twenty patients including 7 probands with autosomal dominant cutis laxa confirm clinical and molecular homogeneity. *Orphanet J Rare Dis* **8**, 36, doi:10.1186/1750-1172-8-36 (2013).

19 Garnai, S. J. *et al.* Variants in myelin regulatory factor (MYRF) cause autosomal dominant and syndromic nanophthalmos in humans and retinal degeneration in mice. *PLoS Genet* **15**, e1008130, doi:10.1371/journal.pgen.1008130 (2019).

20 Siggs, O. M. *et al.* Autosomal dominant nanophthalmos and high hyperopia associated with a C-terminal frameshift variant in MYRF. *Mol Vis* **25**, 527-534 (2019).

21 Scott, A. F. *et al.* Identification of an HMGB3 frameshift mutation in a family with an X-linked colobomatous microphthalmia syndrome using whole-genome and X-exome sequencing. *JAMA Ophthalmol* **132**, 1215-1220, doi:10.1001/jamaophthalmol.2014.1731 (2014).
22 Watson, C. M. *et al.* Deficiency of the myogenic factor MyoD causes a perinatally lethal fetal akinesia. *J Med Genet* **53**, 264-269, doi:10.1136/jmedgenet-2015-103620 (2016).
23 Voronina, V. A. *et al.* Mutations in the human RAX homeobox gene in a patient with anophthalmia and sclerocornea. *Hum Mol Genet* **13**, 315-322, doi:10.1093/hmg/ddh025 (2004).
24 Wu, C. W. *et al.* Phenotype expansion of heterozygous FOXC1 pathogenic variants toward involvement of congenital anomalies of the kidneys and urinary tract (CAKUT). *Genet Med* **22**, 1673-1681, doi:10.1038/s41436-020-0844-z (2020).
25 Slot, E. *et al.* Alveolar capillary dysplasia with misalignment of the pulmonary veins: clinical, histological, and genetic aspects. *Pulm Circ* **8**, 2045894018795143, doi:10.1177/2045894018795143 (2018).
26 Hayashi, Y., Harada, Y. & Harada, H. Myeloid neoplasms and clonal hematopoiesis from the RUNX1 perspective. *Leukemia* **36**, 1203-1214, doi:10.1038/s41375-022-01548-7 (2022).
27 Boutoleau-Bretonniere, C. *et al.* A phenotype of atypical apraxia of speech in a family carrying SQSTM1 mutation. *J Alzheimers Dis* **43**, 625-630, doi:10.3233/JAD-141512 (2015).
28 Bucelli, R. C. *et al.* SQSTM1 splice site mutation in distal myopathy with rimmed vacuoles. *Neurology* **85**, 665-674, doi:10.1212/WNL.0000000000001864 (2015).
29 Haack, T. B. *et al.* Absence of the Autophagy Adaptor SQSTM1/p62 Causes Childhood-Onset Neurodegeneration with Ataxia, Dystonia, and Gaze Palsy. *Am J Hum Genet* **99**, 735-743, doi:10.1016/j.ajhg.2016.06.026 (2016).
30 Muto, V. *et al.* Biallelic SQSTM1 mutations in early-onset, variably progressive neurodegeneration. *Neurology* **91**, e319-e330, doi:10.1212/WNL.0000000000005869 (2018).
31 Tucker, E. J. The Genetics and Biology of FOXL2. *Sex Dev*, 1-10, doi:10.1159/000519836 (2021).
32 Beysen, D. *et al.* Identification of 34 novel and 56 known FOXL2 mutations in patients with Blepharophimosis syndrome. *Hum Mutat* **29**, E205-219, doi:10.1002/humu.20819 (2008).
33 Yang, L., Li, T. & Xing, Y. Identification of a novel FOXL2 mutation in a single family with both types of blepharophimosis-ptosis-epicanthus inversus syndrome. *Mol Med Rep* **16**, 5529-5532, doi:10.3892/mmr.2017.7226 (2017).
34 Weese-Mayer, D. E., Rand, C. M., Zhou, A., Carroll, M. S. & Hunt, C. E. Congenital central hypoventilation syndrome: a bedside-to-bench success story for advancing early diagnosis and treatment and improved survival and quality of life. *Pediatr Res* **81**, 192-201, doi:10.1038/pr.2016.196 (2017).
35 Loscocco, G. G. & Vannucchi, A. M. Role of JAK inhibitors in myeloproliferative neoplasms: current point of view and perspectives. *Int J Hematol* **115**, 626-644, doi:10.1007/s12185-022-03335-7 (2022).
36 Williamson, K. A., Yates, T. M. & FitzPatrick, D. R. in *GeneReviews((R))* (eds M. P. Adam *et al.*) (1993).
37 Giusti, F., Marini, F. & Brandi, M. L. in *GeneReviews((R))* (eds M. P. Adam *et al.*) (1993).
38 Bunn, K. J. *et al.* Mutations in DVL1 cause an osteosclerotic form of Robinow syndrome. *Am J Hum Genet* **96**, 623-630, doi:10.1016/j.ajhg.2015.02.010 (2015).
39 White, J. *et al.* DVL1 frameshift mutations clustering in the penultimate exon cause autosomal-dominant Robinow syndrome. *Am J Hum Genet* **96**, 612-622, doi:10.1016/j.ajhg.2015.02.015 (2015).
