# Supplementary material for: Aberrant phase separation and nucleolar dysfunction in rare genetic diseases
Source: Nature. 2023 Feb 8;614(7948):564–71. doi: 10.1038/s41586-022-05682-1 (PMC9931588; doi:10.1038/s41586-022-05682-1)
Supplement: Supplementary file 2 — Reporting Summary [file 41586_2022_5682_MOESM2_ESM.pdf]

nature portfolio

Corresponding author(s): Malte Spielmann, Denise Horn, Denes Hnisz

Last updated by author(s): Nov 13, 2022

Reporting Summary

Nature Portfolio wishes to improve the reproducibility of the work that we publish. This form provides structure for consistency and transparency in reporting. For further information on Nature Portfolio policies, see our Editorial Policies and the Editorial Policy Checklist.

Statistics

For all statistical analyses, confirm that the following items are present in the figure legend, table legend, main text, or Methods section.

n/a	Confirmed	
☐	☒	The exact sample size (n) for each experimental group/condition, given as a discrete number and unit of measurement
☒	☐	A statement on whether measurements were taken from distinct samples or whether the same sample was measured repeatedly
☐	☒	The statistical test(s) used AND whether they are one- or two-sided<br>*Only common tests should be described solely by name; describe more complex techniques in the Methods section.*
☒	☐	A description of all covariates tested
☒	☐	A description of any assumptions or corrections, such as tests of normality and adjustment for multiple comparisons
☐	☒	A full description of the statistical parameters including central tendency (e.g. means) or other basic estimates (e.g. regression coefficient) AND variation (e.g. standard deviation) or associated estimates of uncertainty (e.g. confidence intervals)
☐	☒	For null hypothesis testing, the test statistic (e.g. F, t, r) with confidence intervals, effect sizes, degrees of freedom and P value noted<br>*Give P values as exact values whenever suitable.*
☒	☐	For Bayesian analysis, information on the choice of priors and Markov chain Monte Carlo settings
☒	☐	For hierarchical and complex designs, identification of the appropriate level for tests and full reporting of outcomes
☐	☒	Estimates of effect sizes (e.g. Cohen's d, Pearson's r), indicating how they were calculated

Our web collection on statistics for biologists contains articles on many of the points above.

Software and code

Policy information about availability of computer code

Data collection	Data from fluorescence microscopy was acquired with Zen Black v3.2 (Zeiss).
Data analysis	Facial phenotyping and composite mask creation was performed using Face2Gene v.20.1.4 (face2gene.com). Microscopy data was analyzed using ZenBlue 3.2, except v 3.4 was used in puromycylation experiment, as described in the methods. Protein structure predictions were performed using in-house implementation of AlphaFold 2.0.0. Visualization with ChimeraX 1.5.<br><br>Following tools were used in building variant catalogue: Metapredict v1.51, Ensembldb v2.22.0, BEDtools v2.30.0, VEP v104, CADD v1.6, pVACtools v3.1.0, localCIDER 0.1.18, biopython v1.79, NoD v1.0.0<br><br>Custom code is available at GitHub:<br>https://github.com/hniszlab/HMGB1_2022<br>https://github.com/alexpmagalhaes/IDR-variant-catalog

For manuscripts utilizing custom algorithms or software that are central to the research but not yet described in published literature, software must be made available to editors and reviewers. We strongly encourage code deposition in a community repository (e.g. GitHub). See the Nature Portfolio guidelines for submitting code & software for further information.

Data

Policy information about availability of data

All manuscripts must include a data availability statement. This statement should provide the following information, where applicable:

- Accession codes, unique identifiers, or web links for publicly available datasets
- A description of any restrictions on data availability
- For clinical datasets or third party data, please ensure that the statement adheres to our policy

Patient consent did not cover the release of personal sequence information other than the causative pathogenic variants. Therefore, the pathogenic variants are disclosed in this paper, but whole genome and exome sequencing data cannot be made publicly available for reasons of data protection and patient privacy. Statistical source data are made available with this paper. Primer sequences are listed in Supplementary Table 6. Plasmids are available at Addgene. Circular Dichroism (CD) spectra were deposited at the PCDDB under the accession IDs: CD0006401000, CD0006401001, CD0006404000, CD0006404001.

Following databases were used in building the variant catalogue:
GENCODE GRCh38.p13 Release 41 https://www.gencodegenes.org/human/
MobiDB 4.1.0 https://mobidb.bio.unipd.it/
Ensembldb v104 https://bioconductor.org/packages/release/bioc/html/ensembldb.html
Clinvar 1.64 https://www.ncbi.nlm.nih.gov/clinvar/
COSMIC v95 https://cancer.sanger.ac.uk/cosmic
dbDNP from May 26, 2020 https://www.ncbi.nlm.nih.gov/snp/
1000 genomes from 2021-11-20 https://www.internationalgenome.org/data

Field-specific reporting

Please select the one below that is the best fit for your research. If you are not sure, read the appropriate sections before making your selection.

☒ Life sciences ☐ Behavioural & social sciences ☐ Ecological, evolutionary & environmental sciences

For a reference copy of the document with all sections, see nature.com/documents/nr-reporting-summary-flat.pdf

Life sciences study design

All studies must disclose on these points even when the disclosure is negative.

Sample size	Required family number for new disease gene establishment is 2, number of unrelated families included in our studies is 5.<br><br>No statistical methods were used to predetermine sample sizes. Sample sizes are indicated in figures, legends or in the methods. For droplet assays, 5 – 10 independent fields of view were imaged per condition for each experiment based on current practices in the field (Sabari et al. 2018, Boija et al. 2018) and performed independently total 3 times.
Data exclusions	No data were excluded
Replication	Genetic test results were confirmed by an independent method (WGS by bidirectional Sanger sequencing; chromosomal microarray analysis by RT-qPCR). Genetic tests were also performed on unaffected controls.<br><br>Results from biochemistry and cellular experiments were replicated across multiple experiments with similar results. Numbers of replicates are indicated in figures, figure legends or in the methods.
Randomization	N.a. for participants. In cell culture, wells were randomly assigned for transfections and control or treatment groups. For microscopy, image fields were randomly selected while avoiding cells with highest expression to avoid saturation of EGFP channel.
Blinding	Blinding of patient phenotypical data is impossible, as this is required for genome data interpretation and blinding was not performed. Investigators were not blinded during data acquisition from biochemistry and cellular experiments. Differences between wild type and mutant samples were so apparent in microscopy experiments that blinding would not have been feasible. Analytical pipelines for experiments were uniform across samples, allowing unbiased analysis of data.

Reporting for specific materials, systems and methods

We require information from authors about some types of materials, experimental systems and methods used in many studies. Here, indicate whether each material, system or method listed is relevant to your study. If you are not sure if a list item applies to your research, read the appropriate section before selecting a response.

Materials & experimental systems

n/a	Involved in the study
☐	☒ Antibodies
☐	☒ Eukaryotic cell lines
☒	☐ Palaeontology and archaeology
☒	☐ Animals and other organisms
☐	☒ Human research participants
☒	☐ Clinical data
☒	☐ Dual use research of concern

Methods

n/a	Involved in the study
☒	☐ ChIP-seq
☐	☒ Flow cytometry
☒	☐ MRI-based neuroimaging

Antibodies

Antibodies used

HP1a (1:500, Cell signaling Cat# 2616S) MED1 (1:500, Abcam Cat# ab64965) RNAPII (1:500, Abcam, Cat# ab26721) NPM1 (1:250, Thermo Fisher Scientific, Cat# 32-5200) FIB1 (1:100, Santa Cruz Cat# sc-374022) SC-35 (1:200, Sigma-Aldrich Cat# S4045), HMGB1 (1:1000 Sigma-Aldrich Cat# H9664), EGFP (1:1000, Invitrogen Cat# A-11122), anti-Puromycin (1:1000, Sigma Aldrich MABE343), anti-GFP (1:2000, Abcam ab13970), Alexa Fluor 647 donkey anti-mouse (1:1000, Jackson Immuno Research, Cat# 715-605-150), Alexa Fluor 647 donkey anti-rabbit (1:1000, Jackson Immuno Research, Cat# 711-605-152), HRP-Donkey anti-Rabbit IgG (1:2000, Jackson Immuno Research, Cat# 711-035-152), 488-anti-chicken (1:250, Jackson Immuno Research, Cat# 703-545-155), 647-anti-mouse (1:250, Jackson Immuno Research, Cat #715-605-151)

Validation

All antibodies are verified by manufacturer, described to function in human cells in intended applications (immunofluorescence or western blot) and used in numerous publications. Lists for publications are available on manufacturer's websites:

HP1a: https://www.cellsignal.com/products/primary-antibodies/hp1a-antibody/2616
MED1: https://www.abcam.com/trap220med1-antibody-ab64965.html
RNAPII: https://www.abcam.com/rna-polymerase-ii-ctd-repeat-ysptsps-antibody-chip-grade-ab26721.html
NPM1: https://www.thermofisher.com/antibody/product/NPM1-Antibody-clone-FC-61991-Monoclonal/32-5200
FIB1: https://www.scbt.com/p/fibrillarin-antibody-g-8
SC-35: https://www.sigmaaldrich.com/DE/en/product/sigma/s4045
HMGB1: https://www.sigmaaldrich.com/DE/en/product/sigma/h9664
EGFP: https://www.thermofisher.com/antibody/product/GFP-Antibody-Polyclonal/A-11122
Puromycin: https://www.sigmaaldrich.com/DE/en/product/mm/mabe343
GFP: https://www.abcam.com/gfp-antibody-ab13970.html

Statement for knock-out verification for NPM1 antibody is provided on manufacturer's website: "This Antibody was verified by Knockdown to ensure that the antibody binds to the antigen stated.":
https://www.thermofisher.com/antibody/product/NPM1-Antibody-clone-FC-61991-Monoclonal/32-5200

HMGB1 antibody is provided with validation statement (Enhanced validation, independent):
https://www.sigmaaldrich.com/DE/en/product/sigma/h9664

https://www.sigmaaldrich.com/DE/en/technical-documents/technical-article/protein-biology/immunohistochemistry/antibody-enhanced-validation

EGFP antibody is provided with Advanced verification statement: "This Antibody was verified by Relative expression to ensure that the antibody binds to the antigen stated."
https://www.thermofisher.com/antibody/product/GFP-Antibody-Polyclonal/A-11122

No in-house validations for antibodies were performed.

Eukaryotic cell lines

Policy information about cell lines

Cell line source(s)

HEK293T (ATCC CRL-3216), MCF7 (ATCC HTB-22), HCT116 (ATCC CCL-247), U2OS-2-6-3 cell line was received from Richard Young lab (Zamudio et al. 2019), U2OS cells from ATCC (HTB-96) were used for generating Doxycycline-inducible HMGB1-expressing cell lines.

Authentication

Identity of cell lines has been validated using morphological characteristics, but they were not authenticated.

Mycoplasma contamination

All cell lines were tested negative for mycoplasma using PCR Mycoplasma testkit II (A8994, AppliChem).

Commonly misidentified lines
(See ICLAC register)

None used.

Human research participants

Policy information about studies involving human research participants

Population characteristics	5 participants have (de novo) heterozygous frameshifts in HMGB1's acidic tail, 1 individual has a de novo heterozygous deletion of HMGB1. Individuals originate from different populations (Russian, Iranian, German, Hong-Kong-Chinese, Venezuelan) and are unrelated. 3 are male 3 are female. Ages reach from 21 weeks of gestation to 29 years. 5 individuals are diagnosed with BPTAS, one is diagnosed with neurodevelopmental delay.
Recruitment	Individuals were recruited during routine patient care in 5 genetics departments (Berlin, Kiel, Nuremberg, Schwerin, Hong Kong). Potentially affected fetuses could not be systematically screened, thus the frequency and severity spectrum of BPTAS in unborn individuals remains unknown.
Ethics oversight	The study was approved by the ethical review board of the Charité - Universitätsmedizin Berlin (EA2/087/15).

Note that full information on the approval of the study protocol must also be provided in the manuscript.

Flow Cytometry

Plots

Confirm that:

☒ The axis labels state the marker and fluorochrome used (e.g. CD4-FITC).

☒ The axis scales are clearly visible. Include numbers along axes only for bottom left plot of group (a 'group' is an analysis of identical markers).

☒ All plots are contour plots with outliers or pseudocolor plots.

☒ A numerical value for number of cells or percentage (with statistics) is provided.

Methodology

Sample preparation	Cells were collected for FACS from 6-well plates with TrypLE (12604013, Gibco), washed once with cell culture medium, once with PBS, then resuspended to FACS buffer (2 % FBS, 0.5 mM EDTA in PBS) and transferred to FACS tube (#352235, Falcon) through 35 μm nylon mesh.
Instrument	FACS Aria II Flow Cytometer (BD Biosciences)
Software	BD FACS Diva v.6.1.3
Cell population abundance	The percentage of EGFP positive population in transfected cells ranged from 20 to 30 % between replicate experiments.
Gating strategy	To select appropriate gating for EGFP positive cells, untransfected cells were used as a negative control.

☒ Tick this box to confirm that a figure exemplifying the gating strategy is provided in the Supplementary Information.
