# Supplementary material for: Aberrant phase separation and nucleolar dysfunction in rare genetic diseases
Source: Nature. 2023 Feb 8;614(7948):564–71. doi: 10.1038/s41586-022-05682-1 (PMC9931588; doi:10.1038/s41586-022-05682-1)
Supplement: Supplementary file 4 — Clinical characteristics of affected individuals with BPTAS. [file 41586_2022_5682_MOESM4_ESM.docx]

## **Supplementary Table 1: Clinical characteristics of individuals with BPTAS**

|  | I1 | I2 | I3  Shafeghati et al. | I4 | I5 | Baraitser et al.  (pat.1) | Baraitser et al.  (pat.2) | Bernardi et al. | Faravelli et al. | Wechsler et al. | Pierson et al.  (pat.1) | Pierson et al.  (pat.2=  father) | Olney et al. | Total |
| --- | --- | --- | --- | --- | --- | --- | --- | --- | --- | --- | --- | --- | --- | --- |
| Sex | M | F | F | M | F | M | M | F | M | F | M | M | M |  |
| Age at last assessment | 9 months | 29 years | 11 months | 12 weeks | 21rst week of gestation | 7 months | 6 years | 12 months | < 12 months | 10 months | 12 days | 36 years | 1rst day |  |
| **Skeletal phenotype** |  |  |  |  |  |  |  |  |  |  |  |  |  |  |
| Shortened and malformed lower limbs with tibial aplasia/hypoplasia | + | + | + | + | + | + | + | + | + | + | + | na | + | 12/12 |
| Preaxial polydactyly of the feet | + | + | + | + | + | + | + | + | + | + | + | + | + | 13/13 |
| Shortening of radius/ulna | + | + | + | na | + | + | + | + | + | + | + | - | + | 11/12 |
| Brachyphalangy of fingers | + | + | + | + | + | + | + | + | + | + | + | na | + | 12/12 |
| Syndactyly of fingers/toes | + | + | + | + | + | + | + | + | + | + | + | na | + | 12/12 |
| Hypoplastic/absent/dislocated nails, enlargement of distal phalanges in the dorso-ventral axis | + | + | + | na | + | + | + | + | + | + | + | - | + | 11/12 |
| Short middle phalanges and proximal phalanges of the thumb | + | + | + | + | + | + | + | + | + | + | + | na | na | 11/11 |
| Hypoplastic iliac wings | + | + | + | - | + | + | + | + | + | + | + | na | + | 11/12 |
| Contractures/pterygia of joints | + | + | + | + | + | + | + | + | + | + | + | na | + | 12/12 |
| **Craniofacial features** |  |  |  |  |  |  |  |  |  |  |  |  |  |  |
| Microcephaly | + | + | + | + | + | + | + | + | + | + | + | - | + | 12/13 |
| Malformed ears/microtia | + | + | + | + | + | + | + | + | + | + | + | + | + | 13/13 |
| Blepharophimosis/wide set eyes | + | + | + | + | + | na | + | + | + | + | + | + | + | 12/12 |
| **Neurological features** |  |  |  |  |  |  |  |  |  |  |  |  |  |  |
| Developmental delay | + | + | na | na | na | na | +, only  motor | +, only  motor | +, only  motor | +, only  motor | na | na | na | 6/6 |
| Hearing impairment | + | + | + | - | na | na | + | + | na | + | na | + | na | 7/8 |
| **Other features** |  |  |  |  |  |  |  |  |  |  |  |  |  |  |
| Genitourinary anomalies | + | na | + | + | + | + | + | + | + | + | + | - | na | 10/11 |

na: Data not available
